# An Exploratory Study on Vectorcardiographic Identification of the Site of Origin of Focally Induced Premature Depolarizations in Horses, Part II: The Ventricles

**DOI:** 10.3390/ani12050550

**Published:** 2022-02-23

**Authors:** Glenn Van Steenkiste, Tammo Delhaas, Ben Hermans, Lisse Vera, Annelies Decloedt, Gunther van Loon

**Affiliations:** 1 Equine Cardioteam, Department of Internal Medicine, Reproduction and Population Medicine, Faculty of Veterinary Medicine, Ghent University, 9820 Merelbeke, Belgium; lissevera@hotmail.be (L.V.); annelies.decloedt@ugent.be (A.D.); gunther.vanloon@ugent.be (G.v.L.); 2 Department of Biomedical Engineering, CARIM School for Cardiovascular Diseases, Maastricht University, 6200 MD Maastricht, The Netherlands; tammo.delhaas@maastrichtuniversity.nl (T.D.); ben.hermans@maastrichtuniversity.nl (B.H.)

**Keywords:** focal, ventricular tachycardia, QRS complex, electrophysiology, electrocardiography

## Abstract

**Simple Summary:**

Ventricular arrhythmias occur commonly in horses. Knowledge on the origin of ventricular arrhythmias is essential for proper treatment. Former studies in horses showed contradictory results regarding the diagnostic value of 12-lead electrocardiography and vectorcardiography due to the anatomical differences in horses compared to humans and small animals. As a consequence, no standardized approach is available for electrocardiography electrode configurations in horses. The current study investigated whether the anatomical origin of experimentally induced ventricular premature depolarizations in horses could be differentiated based upon spherical statistics of the vectorcardiography characteristics. Vectorcardiography shows the magnitude and direction of the cardiac electrical forces in three dimensions. The vectorcardiogram was recorded in seven horses under general anesthesia while right and left ventricular pacing was performed from inside the heart. Using spherical statistics, it could be shown that pacing induces significantly different initial and maximum electrical axes between different locations and between pacing and normal sinus rhythm. The current approach could be used in clinical patients to identify the origin of ventricular arrhythmias without the need for invasive studies. The technique could also be used in other species for which a standardized electrocardiogram electrode configuration is not available.

**Abstract:**

In human cardiology, the anatomical origin of ventricular premature depolarizations (VPDs) is determined by the characteristics of a 12-lead electrocardiogram (ECG). Former studies in horses had contradictory results regarding the diagnostic value of the 12-lead ECG and vectorcardiography (VCG), which results were attributed to the different cardiac conduction system in this species. The objective of this study was to determine if the anatomical origin of pacing-induced VPDs could be differentiated in horses based upon VCG characteristics. A 12-lead ECG was recorded in seven horses under general anesthesia while right and left ventricular endomyocardial pacing was performed (800–1000 ms cycle length) at the apex, mid and high septum and mid and high free wall, and at the right ventricular outflow tract. Catheter positioning was guided by 3D electro-anatomical mapping and echocardiography. A median complex, obtained from four consecutive complexes, was calculated for each pacing location and sinus rhythm. The VCG was calculated from the 12-lead ECG-derived median complexes using custom-made algorithms and was used to determine the initial and maximum electrical axes of the QRS complex. An ANOVA for spherical data was used to test if VCGs between each paced location and between pacing and sinus rhythm were significantly (*p* < 0.05) different. The model included the radius, azimuth and elevation of each electrical axis. Pacing induced significantly different initial and maximum electrical axes between different locations and between pacing and sinus rhythm. The current results suggest that VCG is a useful technique to identify the anatomical origin of ventricular ectopy in horses.

## 1. Introduction

The use of a 12-lead ECG has proven to be a very effective method for identification and differentiation of the underlying mechanisms of ventricular arrhythmias as well as for preparticipation screening for cardiovascular diseases in human athletes in order to reduce the casualties of sudden cardiac death [1]. Because knowledge from human cardiology cannot be directly extrapolated to horses due to their different ventricular conduction system [2], preparticipation screening of equine athletes with their high prevalence of ventricular arrhythmias is cumbersome [3]. Former studies in horses also had contradictory results regarding the diagnostic value of the 12-lead ECG for localization of premature depolarizations or prediction of the associated risk of ventricular arrhythmias [4,5,6,7], possibly partly due to suboptimal electrode positioning [8,9,10]. Recent studies showed that a 12-lead ECG adds value in equine cardiology if the lead system is properly adjusted to the anatomical position of the equine heart [11,12,13].

Nowadays, the vector cardiogram (VCG) has regained interest in human medicine because of more easy-to-use equipment [14]. The VCG provides additional insight that remains unexplored in the 12-lead ECG, such as the true QRS complex direction and amplitude in three dimensions and the spatial angle between the initial and maximum electrical axis of the QRS complex [14,15]. The VCG can be used to increase the power of statistical analyses by decreasing the number of independent variables because calculating the VCG from the 12-lead ECG offers a reduction in the number of independent leads from eight to three, with only minimal information loss [16].

The aim of this exploratory study was, first, to determine if it is possible to differentiate induced ventricular premature depolarizations originating from specific anatomical areas using a 12-lead ECG-derived VCG and, second, to differentiate them from sinus beats.

## 2. Materials and Methods

A cross-sectional analytic study design was used. This study was approved by the ethical committee of the Faculty of Veterinary Medicine, Ghent University (Merelbeke, Belgium) (EC 2016/35) and animal care was according to their guidelines. Seven warmblood geldings aged 12.5 (5–20) years (median (range)) with height 163 cm (155–179 cm) at the withers and mass 548 kg (420–706 kg) were used. Four horses were owned by the Faculty of Veterinary Medicine, whereas three horses were donated for scientific research followed by euthanasia because of orthopedic problems. Horses were included in the study if auscultation, biochemistry (electrolytes and cardiac troponin I), echocardiography and ECG were normal. Data from the currently described study were obtained at the same time as those from a previously described study [12].

### 2.1. Electrophysiological Study

**Figure 1:**
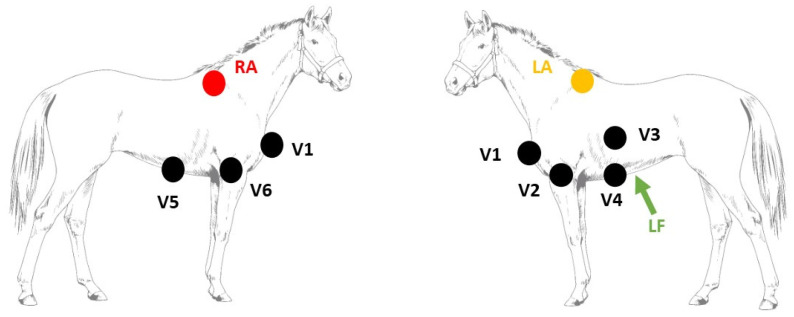
Electrode placement to record the 12-lead electrocardiogram. Abbreviations: LA, left arm electrode; LF, left foot electrode; RA, right arm electrode; V, precordial electrode.

The study was performed under general anesthesia. Prior to pacing, a complete endomyocardial 3D electro-anatomical map of the ventricles (RHYTHMIA v1.4, Boston Scientific, Diegem, Belgium) was created, as described elsewhere [17]. The 3D electro-anatomical map was used as a reference to navigate the mapping/pacing catheter (INTELLAMAP ORION, Boston Scientific, Diegem, Belgium) inside the ventricles to specific anatomical locations. Simultaneously performed echocardiography (Vivid 7, GE Healthcare, Diegem, Belgium) served as an additional confirmation of the pacing location. Suprathreshold ventricular pacing was performed at 800 or 1000 ms cycle length (EPS320, MicroPace EP Inc., Santa Ana, CA, USA) at the following right and left ventricular locations: apex, mid and high septum as well as mid and high lateral ventricular free wall. In addition, pacing was performed at the free wall of the right ventricular outflow tract below the pulmonary valve. At each location, twenty pacing-induced VPDs were recorded with a 12-lead ECG (LABSYSTEM Pro v2.6, Boston Scientific, Diegem, Belgium) with the following electrode configuration [11] (Figure 1): left and right arm electrode on the left and right dorsal spina scapula, respectively; left foot electrode at the abdominal midline caudal to the xiphoid process; precordial leads on the manubrium sterni (V1), on the ventral part of the left (V2) and right (V6) triceps muscle, in the left 6th intercostal space at the level of the shoulder joint (V3) and elbow joint (V4) and in the right 6th intercostal space at the level of the elbow joint (V5). The VCG axes were calculated from the 12-lead ECG using the following equations:

(1) $$X=\frac{V2+V4}{2}-\frac{V5+V6}{2}$$

(2) $$Y=\frac{V4+V5}{2}-\frac{V2+V6\;}{2}\;Z=-aVF$$

with X the right–left, Y the cranial–caudal and Z the ventral–dorsal leads [18].

### 2.2. Data Analysis

**Figure 2:**
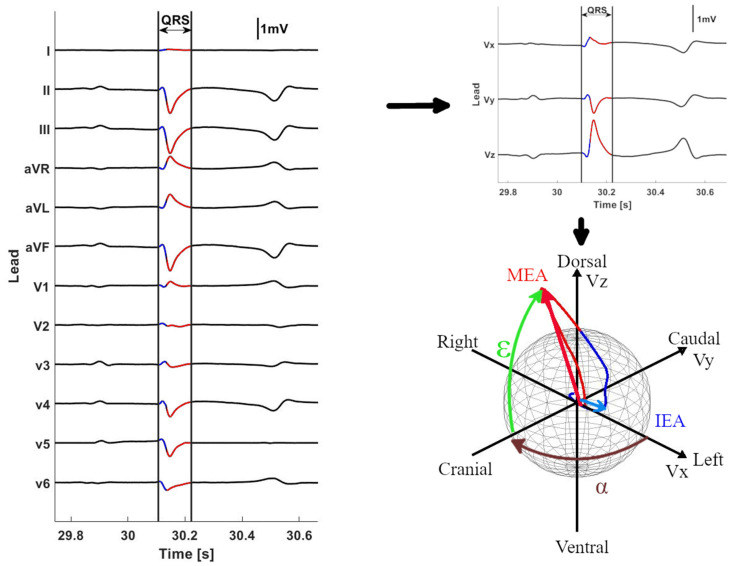
Example of the analysis of a QRS complex originating from the right ventricular outflow tract. First, the QRS complex (red) was manually identified on the 12-lead ECG; after which, the initial QRS signal (blue) was automatically selected within the first 40 ms after onset. After selecting the QRS complex, the VCG axes were derived from the 12-lead ECG with Vx right–left, Vy cranial–caudal and Vz ventral–dorsal. Next, the spherical coordinates were calculated from the VCG. If a local maximum could be found within the first 40 ms, this was selected as the initial electrical axis (IEA), but if no local maximum could be found, the IEA was defined as the coordinates with the largest radius within the first 40 ms. The maximum (MEA) electrical axis was automatically defined as the coordinates with the largest radius within entire duration of the QRS complex. The IEA and MEA are shown by the blue and red arrows on the sphere, respectively. For each electrical axis, the azimuth (α) and elevation (ε) were calculated as shown on the sphere. The intersection of the sphere and the electrical axis is projected on the Lambert azimuthal equal-area plots of Figure 3 and Figure 4.

Analyses were performed using custom-made programs (MATLAB R2018b, Mathworks, Einthoven, The Netherlands). In order to improve the signal-to-noise ratio, a median complex was calculated. This median complex is the composite of several consecutive complexes that were aligned and of which the median value was taken for each timepoint of the cardiac cycle. The median complex was calculated for each paced location and sinus rhythm (SR). Each complex was manually labeled by a single observer. Unanalyzable complexes due to noise and artifacts and complexes with a deviant morphology were excluded. Some recordings only had four consecutive complexes with a stable, noise-free morphology; therefore, the median was calculated out of 4 consecutive complexes with alignment on the maximum absolute value of the QRS. The representative median complex was constructed for each single lead using the median function of MATLAB. The onset and offset of the QRS was manually selected by an observer with experience in equine electrocardiography. The distance between the origin of the spherical coordinate system of the VCG, defined as the onset of the QRS complex, and a point on the QRS vector loop was taken as a measure of the amplitude and can be calculated using the Euclidean $\left|\left|V_{m}\right|\right|=\sqrt[{2}]{X^{2}+Y^{2}+Z^{2}}$. A local peak amplitude on the vector amplitude within 40 ms from the onset of the QRS complex was considered to be indicative of the spatial initial electrical axis (IEA), whereas the maximum amplitude of the QRS complex was considered to be the maximum electrical axis (MEA; Figure 2). If no local peak amplitude could be found within the first 40 ms, the maximum vector amplitude within the first 40 ms was considered the IEA. The size and direction of each IEA and MEA were automatically detected and expressed by the vector amplitude, azimuth (clock-wise estimated angle between the projected vector in the X-Y plane and the positive *X*-axis) and elevation (angle between vector and X-Y plane with positive values denoting dorsally directed values).

### 2.3. Statistical Analysis

All statistics were computed in R (R 3.6.1, R Foundation for Statistical Computing, Vienna, Austria). Results of IEA and MEA are described as mean ± standard deviation and with a 95% confidence interval. For statistical analysis using directional data analytics, the radii of all IEA and MEA coordinates were set to 1 in order to work on a unit sphere and all data points were converted to a cartesian coordinate system similar to Figure 2 [19]. The function fishkent in the Directional package was used to test the null hypothesis that a von Mises–Fisher distribution, in which points are isotopically concentrated around a mean direction (rotational symmetry), fit the data well. Rejection of the null hypothesis (*p* < 0.05) would imply a lack of rotational symmetry; in which case, a Kent distribution would be more appropriate [20]. The Shapiro–Wilk normality test was used to determine if the durations and maximum amplitudes of the QRS loops were derived from a normal distribution. The mean direction and concentration parameter κ of the direction of IEA and MEA were calculated for all datapoints [21]. For each combination of paced locations, and between each pacing location and sinus rhythm, a spherical log-likelihood ANOVA was used to determine if the directions were significantly (*p* < 0.05) different. The ANOVA was followed by a post hoc analysis in order to describe the significant differences between the individual paced locations and SR.

## 3. Results

**Figure 3:**
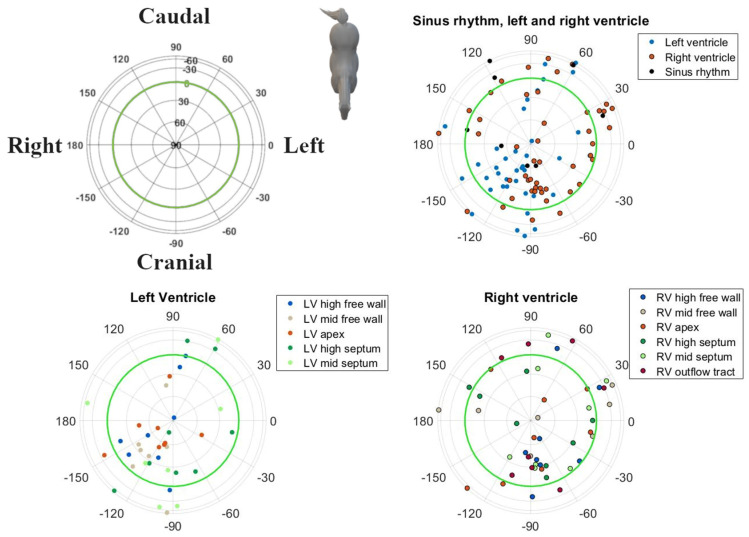
Spatial directions of the initial electrical axis (IEA) of the QRS complex for individual spatial directions of induced VPDs, visualized using a Lambert azimuthal equal-area plot. The spatial direction of the IEA was calculated during the first 40 ms of the QRS complex. Dots inside the green circle represent a dorsal IEA, while outside dots represent a ventral IEA. Left is 0°, caudal is 90° azimuth and dorsal is 90° elevation.

**Figure 4:**
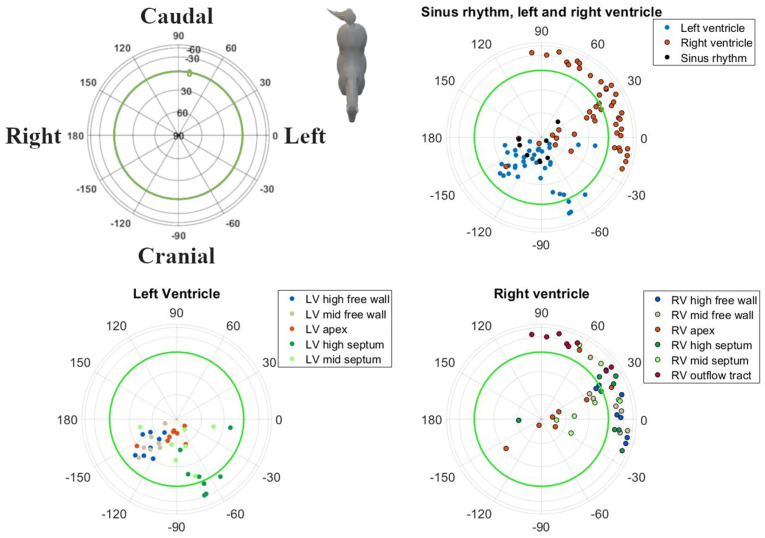
Spatial directions of the maximum electrical axis (MEA) of the QRS complex for individual induced VPDs and SR, visualized with a Lambert azimuthal equal-area plot. The spatial direction of the MEA was calculated over the entire QRS complex. Dots inside the green circle represent a dorsal MEA, while outside dots represent a ventral MEA. Left is 0°, caudal is 90° azimuth and dorsal is 90° elevation.

**Figure 5:**
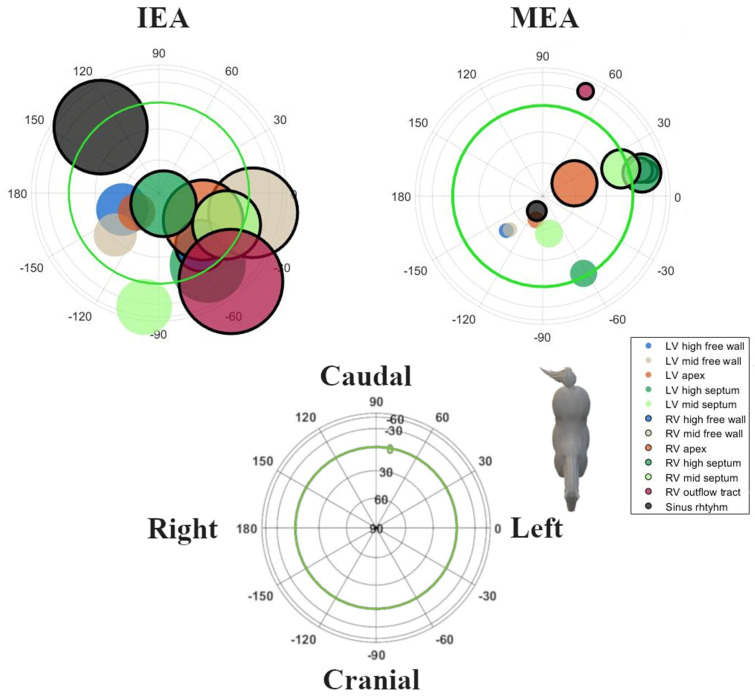
Maximum spatial direction and concentration parameter κ of both the initial (IEA) and maximum (MEA) electrical axes of the QRS complex for induced VPDs and SR, visualized with a Lambert azimuthal equal-area plot. The IEA was defined as the first deflection from the onset of the QRS complex, while the MEA was the largest deflection of the QRS complex. Circles inside the green circle represent a dorsal MEA, while outside circles represent a ventral MEA. Left is 0°, caudal is 90° azimuth and dorsal is 90° elevation. A larger circle indicates a lower κ value and thus a higher spreading of the individual points.

Good-quality ECGs could be recorded during pacing at all locations and during SR. In one horse, pacing data were not available from the RV mid free wall, the LV mid septum and LV mid free wall due to excessive noise on the ECG recordings. Descriptive statistics for the induced VPDs and SR are given in Table 1. The radii followed a normal distribution. Except for the IEAs of SR, the RV outflow tract and RV mid septum, all distributions of the directions within IEAs and all MEAs for SR and each paced location showed rotational symmetry (*p* > 0.05); hence, a von Mises–Fisher distribution was assumed. The directions of the MEA were significantly different (*p* < 0.001) when comparing the combined paced locations against SR. No such significant difference could be found for the IEA (*p* = 0.393). The post hoc test results for individual combinations can be found in Table 2. The mean directions and their concentrations are shown in Figure 5. Due to the large variation in direction of the IEA, only the mean MEA directions are described below. For the individual variations of IEA and MEA, the reader is referred to Figure 3 and Figure 4, in which the individual IEAs and MEAs are shown in the Lambert azimuthal equal-area plot.

The MEA of the SR complexes showed a right craniodorsal direction.

All other VPDs from the LV had a right cranially aimed MEA. Induced VPDs in the LV mid and high free wall had a right craniodorsal MEA. Induced VPDs in the LV septum had a left cranial MEA. A cranially to slightly cranioventrally aimed MEA was noted for VPDs originating from the high LV septum, while the VPDs located at the mid LV septum had a dorsally aimed MEA. Pacing at the LV apex induced a right craniodorsal MEA, similar to SR depolarizations.

As shown in Table 1, pacing at the RV apex induced a left dorsally aimed MEA. All other VPDs originating from the RV had a left caudally aimed MEA. Induced VPDs from the RV outflow tract had a slightly left caudoventrally aimed MEA. Pacing at the RV free wall induced a left, slightly towards caudoventral MEA. Induced VPDs from the mid and high RV septum produced a (left) caudal MEA. High RV septal VPDs had a slightly ventrally aimed MEA.

In general, induced VPDs from the LV had a cranial MEA and VPDs from the RV had a caudal MEA. Significant differentiation can be made between the left and right ventricle based upon the MEA (Table 2), with the exceptions of the LV and RV apex, the high septum and the mid septum.

## 4. Discussion

The current study describes VCG characteristics of pacing-induced QRS complexes in horses in an experimental set-up in which the heart was paced from 11 anatomical locations. Analysis of VCG characteristics enabled distinction of specific anatomical origins of the VPDs as well as differentiation between VPDs and SR.

The IEA showed large variations in directions for each pacing location and for SR. In order to increase the reproducibility of the results, we chose a fully automated annotation of the IEA and MEA. However, it appeared to be difficult to identify a clear initial deflection in many cases due to the explosive depolarization of the ventricles. This led to a large variation in IEA directions and amplitudes and, thus, limited usefulness for differentiation between different pacing locations and SR.

The VCG characteristics during SR were similar to those previously reported for semi-orthogonal lead systems in horses, i.e., they showed a dorsally and cranially directed MEA in the spatial VCG [22,23]. Based upon the MEA, it was difficult to differentiate between pacing locations from the RV (Table 2). This is also clear in Figure 5, where the circles of the RV overlap more than the circles of the LV. Only the RV apex and RV outflow tract can be differentiated from the other RV locations. Previous research by our group has shown that the RV in horses has a more explosive depolarization pattern compared to the LV, which may be due to conduction over the septomarginal trabecula [12]. However, in order for the induced VPDs to achieve a similar depolarization pattern to the RV, as during SR, (some of) the induced VPDs would have had to activate the fast His–Purkinje network. In human medicine, VPDs originating from the RV septum have a shorter QRS duration and lower initial amplitude in leads V1–V3 compared to other RV VPDs. These characteristics are used to differentiate between the RV free wall and septum [24,25]. Pacing in the RV apical region could not be differentiated from the RV septum or free wall, which may also reflect the limitations of the pacing catheter used in the current study. Because of the basket shape of the pacing catheter, no perfect apical stimulation could be achieved. Similarly, the large variation in both IEA and MEA of some induced VPD origins could be explained because the pacing site of the induced VPD was not always exactly the same, especially for RV locations, since they were hard to maintain due to the contraction of the RV. If a VPD was induced more to the right of the RV mid free wall, this would depolarize a larger mass in a left direction and thus cause a left directed IEA/MEA. However, if a VPD was induced more cranially, this would produce a more caudal IEA/MEA while still being induced at the RV mid free wall.

Some VPD clusters appear to be similar on the Lambert azimuthal equal-area plots but are still different due to a difference in radius, which is not visible on the Lambert plot and was not included in the current ANOVA analysis. As can be seen in Table 1, most VPDs showed larger maximum radii of their QRS loops than those in SR. Because QRS complexes in SR follow the most optimal depolarization pathway and depolarize both the LV and RV simultaneously, some cancelling out of the cardiac electric field occurred. One notable exception was the VPDs originating from the LV high septum which also showed small radii because the depolarization-induced cardiac electric fields were also cancelled out due to the central location of the pacing site.

In a previous study on horses, VPDs were induced using mechanical stimulation of the epicardium of the right and left ventricular free walls [10]. Changes in QRS patterns were described using a semi-orthogonal lead system. The directions of the electrical axes of the QRS in the left and right ventricular induced VPDs in the horizontal plane were similar to those seen in the current study. However, our study had the advantage that no specialized VCG recording equipment was necessary to perform the recordings. Indeed, the current VCG could be easily recorded with inexpensive, commercially available 12-lead ECG recording devices from human medicine.

Most studies in human patients describe the QRS morphology of VPDs using the 12-lead ECG [25]. Due to the different anatomical position of the heart, the human electrode configuration cannot be directly copied to that of the horse, thus making a direct comparison between the current study in horses and QRS morphologies described in humans difficult. Since the orthogonal coordinate systems of the VCGs in humans and horses are similar, we advocate the use of VCGs to describe and compare QRS morphologies in and between humans and horses.

### Limitations

Though only a small number of animals was used in the current study, the results showed the added value of 12-lead ECG-derived VCG in equine electrocardiography. Larger studies should be conducted in order to enable the construction of a clinically applicable algorithm for the identification of the anatomical site of origin of VPDs. The current study was conducted in healthy horses. It is known in humans that ventricular activation propagates away from the VPD in a predictable manner in individuals without structural heart disease, while propagation is less predictable in the presence of structural heart disease [25]. The current results might thus not be representative for horses with cardiac disease. In our study, only endocardial pacing was performed. In human patients, QRS morphology might be different for endocardial versus epicardial origins of VPDs [26]. These are attributed to the Purkinje network, which is positioned subendocardially in humans, causing endocardial VPDs to propagate faster compared to epicardial VPDs. In horses, the Purkinje network is more diffusely spread in the ventricular myocardium, and it is thus unclear whether the slower conduction for epicardial VPDs also occurs in horses, since transmural differences in activation times between endo and epicardium are known to be below 5 ms during SR in healthy horses [27,28]. In addition, it is unknown if naturally occurring VPDs enter the His–Purkinje system. The current study used suprathreshold pacing for inducing VPDs, which could have activated the His–Purkinje system due to the higher currents compared to a naturally occurring VPD. As a result, the induced VPDs may have a different morphology compared to naturally occurring VPDs. In addition, all recordings were performed under anesthesia in dorsal recumbency, which may have affected the position of the heart and thus the recorded complexes.

## 5. Conclusions

The current results suggest that VCG is a useful technique to identify the anatomical origin of ventricular ectopy in horses.

## Figures and Tables

**Table 1:** Descriptive statistics of the electrical axes’ distribution for sinus rhythm as well as the different induced ventricular premature depolarizations. The initial electrical axis was measured as the local maximum radius during the first 40 ms of the QRS complex. If no local maximum could be found, the initial electrical axis was defined as the maximum during the first 40 ms of the QRS complex. The maximum electrical axis was measured as the maximum radius over the entire QRS complex. The higher the value of concentration parameter κ, the more concentrated the axis direction. In cases where the electrical axes followed a Kent instead of a von Mises–Fisher distribution, the ovalness parameter ß was given. A higher absolute value of ß indicates a more elliptical (flatter) distribution. Normally distributed values are described as mean ± standard deviation while non-normal distributed values are described as median (range).

Pacing Location	Initial Electrical Axis					Maximum Electrical Axis			
	Azimuth (°)	Elevation (°)	Radius (mV)	Κ	ß	Azimuth (°)	Elevation (°)	Radius (mV)	κ
Sinus rhythm	131 ± 97	3 ± 54	0.1 ± 0.1	0.8	−2.57	−109 ± 44	65 ± 7	1.5 ± 0.6	13.5
Left ventricle									
Apex	−140 ± 61	64 ± 25	0.1 ± 0.1	3.4		−99 ± 29	61 ± 21	2.0 ± 1.0	18.0
High free wall	−155 ± 88	53 ± 29	0.1 [0.0–2.2]	1.8		−140 ± 11	39 ± 11	2.0 ± 0.6	31.1
High septum	−55 ± 81	4 ± 43	0.1 ± 0.1	0.9		−60 ± 22	8 ± 23	1.3 ± 0.5	6.7
Mid free wall	−136 ± 46	33 ± 39	0.2 ± 0.1	2.6		−136 ± 10	47 ± 16	3.2 ± 0.8	23.0
Mid septum	−97 ± 81	−37 ± 43	0.0 [0.0–0.4]	1.5		−78 ± 42	48 ± 17	1.9 ± 0.5	5.4
Right ventricle									
Outflow tract	−50 ± 112	−35 ± 32	0.1 ± 0.1	0.8	−3.25	69 ± 19	−34 ± 9	1.8 ± 0.4	18.6
Apex	−31 ± 95	42 ± 42	0.1 ± 0.1	0.7		3 ± 62	34 ± 39	1.9 ± 0.7	2.4
High free wall	−51 ± 56	26 ± 33	0.2 ± 0.1	1.9		16 ± 15	−23 ± 18	2.0 ± 0.4	11.4
High septum	−66 ± 123	80 ± 21	0.1 [0.0–0.6]	1.1		11 ± 20	−20 ± 17	1.8 ± 0.3	12.4
Mid free wall	−11 ± 94	18 ± 54	0.1 ± 0.1	0.6		20 ± 20	−15 ± 20	1.7 ± 0.4	7.9
Mid septum	−25 ± 84	−35 ± 32	0.1 [0.0–0.6]	1.4	2.31	16 ± 26	14 ± 39	1.6 ± 0.6	3.4

**Table 2:** Contingency table with the significant results for the post hoc analysis of the ANOVA for each combination of induced ventricular premature depolarizations and each combination of induced ventricular premature depolarizations and sinus rhythm. The ANOVA was conducted based upon the initial electrical axis and maximum electrical axis calculated from each QRS complex. A *p* < 0.05 is considered a significant difference and is shown in bold.

	Left Ventricle					Right Ventricle					
	apex	hfw	hsep	mfw	msep	apex	hfw	hsep	mfw	msep	OT
*IEA*											
LV hfw	0.073										
LV hsep	**0.032**	**0.044**									
LV mfw	0.079	0.083	0.068								
LV msep	**0.032**	0.060	0.083	0.068							
RV apex	0.083	0.083	0.083	0.083	0.083						
RV hfw	0.080	0.082	0.083	0.081	0.083	0.083					
RV hsep	0.083	0.083	0.083	0.083	0.083	0.083	0.083				
RV mfw	0.083	0.083	0.083	0.083	0.083	0.083	0.083	0.083			
RV msep	0.083	0.083	0.083	0.083	0.083	0.083	0.083	0.083	0.083		
RV OT	0.083	0.083	0.083	0.083	0.083	0.083	0.083	0.083	0.083	0.083	
SR	0.083	0.083	0.083	0.083	0.083	0.083	0.083	0.083	0.083	0.083	0.083
*MEA*											
LV hfw	**<0.001**										
LV hsep	**<0.001**	**<0.001**									
LV mfw	**0.007**	0.083	**<0.001**								
LV msep	0.079	**0.015**	**0.007**	**0.028**							
RV apex	0.054	**0.007**	**0.009**	**0.013**	0.067						
RV hfw	**<0.001**	**<0.001**	**<0.001**	**<0.001**	**<0.001**	**0.011**					
RV hsep	**<0.001**	**<0.001**	**0.018**	**<0.001**	**0.004**	0.063	0.083				
RV mfw	**<0.001**	**<0.001**	**0.004**	**<0.001**	**0.001**	0.059	0.083	0.083			
RV msep	**0.005**	**<0.001**	**0.033**	**0.001**	**0.032**	0.083	0.083	0.083	0.083		
RV OT	**<0.001**	**<0.001**	**<0.001**	**<0.001**	**<0.001**	**0.020**	**0.019**	0.075	0.029	0.056	
SR	0.083	0.066	**0.007**	0.078	0.083	0.083	**<0.001**	**0.004**	**<0.001**	**0.021**	**<0.001**

Abbreviations: hfw, high free wall; hsep, high septum; *IEA*, initial electrical axis; LV, left ventricle; *MEA*, maximum electrical axis; mfw, mid free wall; msep, mid septum; OT, outflow tract; RV, right ventricle; SR, sinus rhythm.

## Data Availability

Data is available upon reasonable request to the authors.

## References

[1] Harmon K.G., Zigman M., Drezner J.A. (2015). The Effectiveness of Screening History, Physical Exam, and ECG to Detect Potentially Lethal Cardiac Disorders in Athletes: A Systematic Review/Meta-Analysis. J. Electrocardiol..

[2] Hamlin R.L., Smith C.R. (1965). Categorization of Common Domestic Mammals Based upon Their Ventricular Activation Process. Ann. N. Y. Acad. Sci..

[3] Navas de Solis C. (2016). Exercising Arrhythmias and Sudden Cardiac Death in Horses: Review of the Literature and Comparative Aspects. Equine Vet. J..

[4] Martin B.B., Reef V.B., Parente E.J., Sage A.D. (1996). Causes of Poor Performance of Horses during Training, Racing, or Showing: 348 Cases (1992–1996). J. Am. Vet. Med. Assoc..

[5] Ryan N., Marr C.M., Mcgladdery A.J. (2010). Survey of Cardiac Arrhythmias during Submaximal and Maximal Exercise in Thoroughbred Racehorses. Equine Vet. J..

[6] Physick-Sheard P.W., McGurrin M.K.J. (2010). Ventricular Arrhythmias during Race Recovery in Standardbred Racehorses and Associations with Autonomic Activity. J. Vet. Intern. Med..

[7] de Solis C.N. (2020). Ventricular Arrhythmias in Horses: Diagnosis, Prognosis and Treatment. Vet. J..

[8] Muylle E., Oyaert W. (1977). Equine Electrocardiography. The Genesis of the Different Configurations of the “QRS” Complex. Zent. Veterinärmedizin Reihe A.

[9] Pfister R., Seifert-Alioth C., Beglinger R. (1984). Die Bestimmung Des Ursprungsortes Ventrikulärer Extrasystolen Beim Pferd. Schweiz. Arch. Tierheilkd. SAT Fachz. Tierärztinnen Tierärzte.

[10] Hamlin R.L., Smetzer D.L., Smith C.R. (1964). Analysis of QRS Complex Recorded Through a Semiorthogonal Lead System in the Horse. Am. J. Physiol..

[11] Hesselkilde E.M., Isaksen J.L., Petersen B.V., Carstensen H., Jespersen T., Pehrson S., Kanters J.K., Buhl R. (2021). A Novel Approach for Obtaining 12-lead Electrocardiograms in Horses. J. Vet. Intern. Med..

[12] Van Steenkiste G., Vera L., Decloedt A., Schauvliege S., Boussy T., van Loon G. (2020). Endocardial Electro-Anatomic Mapping in Healthy Horses: Normal Sinus Impulse Propagation in the Left and Right Atrium and the Ventricles. Vet. J..

[13] Van Steenkiste G., De Clercq D., Vera L., van Loon G. (2019). Specific 12-Lead Electrocardiographic Characteristics That Help to Localize the Anatomical Origin of Ventricular Ectopy in Horses: Preliminary Data. J. Vet. Intern. Med..

[14] Man S., Maan A.C., Schalij M.J., Swenne C.A. (2015). Vectorcardiographic Diagnostic & Prognostic Information Derived from the 12-Lead Electrocardiogram: Historical Review and Clinical Perspective. J. Electrocardiol..

[15] Kors J.A., van Herpen G., Willems J.L., van Bemmel J.H. (1992). Improvement off Automated Electrocardiographic Diagnosis by Combination of Computer Interpretations of the Electrocardiogram and Vectorcardiogram. Am. J. Cardiol..

[16] Kors J.A., van Herpen G., Macfarlane P.W., van Oosterom A., Pahlm O., Kligfield P., Janse M., Camm J. (2011). Computer Analysis of the Electrocardiogram. Comprehensive Electrocardiology.

[17] Van Steenkiste G., De Clercq D., Boussy T., Vera L., Schauvliege S., Decloedt A., van Loon G. (2020). Three Dimensional Ultra-high-density Electro-anatomical Cardiac Mapping in Horses: Methodology. Equine Vet. J..

[18] Holmes J.R., Else R.W. (1972). Further Studies on a New Lead for Equine Electrocardiography. Equine Vet. J..

[19] Cremers J., Klugkist I. (2018). One Direction? A Tutorial for Circular Data Analysis Using R with Examples in Cognitive Psychology. Front. Psychol..

[20] Paine P.J., Preston S.P., Tsagris M., Wood A.T.A. (2018). An Elliptically Symmetric Angular Gaussian Distribution. Stat. Comput..

[21] Sra S. (2012). A Short Note on Parameter Approximation for von Mises-Fisher Distributions: And a Fast Implementation of I s(x). Comput. Stat..

[22] Muylle E., Oyaert W. (1971). Clinical Evaluation of Cardiac Vectors in the Horse. Equine Vet. J..

[23] Holmes J.R. (1976). Spatial Vector Changes during Ventricular Depolarisation Using a Semi-Orthogonal Lead System-A Study of 190 Cases. Equine Vet. J..

[24] Tada H., Ito S., Naito S., Kurosaki K., Ueda M., Shinbo G., Hoshizaki H., Oshima S., Nogami A., Taniguchi K. (2004). Prevalence and Electrocardiographic Characteristics of Idiopathic Ventricular Arrhythmia Originating in the Free Wall of the Right Ventricular Outflow Tract. Circ. J..

[25] Yamada T. (2019). Twelve-Lead Electrocardiographic Localization of Idiopathic Premature Ventricular Contraction Origins. J. Cardiovasc. Electrophysiol..

[26] Berruezo A., Mont L., Nava S., Chueca E., Bartholomay E., Brugada J. (2004). Electrocardiographic Recognition of the Epicardial Origin of Ventricular Tachycardias. Circulation.

[27] Meyling H.A., Ter Borg H. (1957). The Conducting System of the Heart in Hoofed Animals. Cornell Vet..

[28] Muylle E. (1975). Experimenteel Onderzoek naar het Verloop van de Depolarisatiegolf in het Hart van het Paard: De Genesis van het Electrocardiografisch P- En QRS-Complex. Ph.D. Thesis.

